# Unilateral Head Impulses Training in Uncompensated Vestibular Hypofunction

**DOI:** 10.1155/2017/2145173

**Published:** 2017-01-24

**Authors:** Ana Carolina Binetti, Andrea Ximena Varela, Dana Lucila Lucarelli, Daniel Héctor Verdecchia

**Affiliations:** ^1^Vestibular Argentina Institute, Buenos Aires, Argentina; ^2^Department of Otolaryngology, British Hospital of Buenos Aires, Buenos Aires, Argentina; ^3^Center for Medical Research on Human Movement (CIMMHU), Maimónides University, Buenos Aires, Argentina

## Abstract

The aim of this paper is to report a case of a young woman with unilateral vestibular chronic failure with a poorly compensated vestibuloocular reflex during rapid head rotation. Additionally, she developed migraine symptoms during the treatment with associated chronic dizzy sensations and blurred vision. Her report of blurred vision only improved after she completed a rehabilitation program using fast head impulse rotations towards the affected side for 5 consecutive days. We discuss why we elected this form of treatment and how this method may be useful for different patients.

## 1. Introduction

The vestibuloocular reflex (VOR) allows us to keep our eyes fixed on an object during head motion. A VOR deficit generates a retinal slip that can be perceived by the patient as a jump or movement of the observed object while turning the head. This same retinal slip also can serve, by means of adaptive mechanisms, to stimulate cerebellar neuroplasticity. VOR plasticity is therefore modulated by vestibulocerebellar-cortical microcircuits that are activated by specific exercises [1].

Following unilateral vestibular lesions, the vestibular compensation process makes it possible for angular VOR responses to low acceleration head rotations to return to normal. However, a marked asymmetry may persist in response to high velocity head rotation [2].

The head impulse test (HIT) was first described by Halmagyi and Curthoys in 1988 [3]. The HIT is a valuable clinical method for detecting a unilateral vestibular hypofunction and for identifying the affected canals [4, 5]. In 2009, Weber et al. [6] presented a video-assisted version of the HIT (vHIT) that enabled a graphic record of the VOR deficit in each of the six semicircular canals and a means to measure their recovery [7]. This system also enabled the detection of overt saccades, which are a sign of vestibular hypofunction when they appear after head rotation, and covert saccades, which appear during head rotation and cannot be detected by the human eye in a clinical examination but rather can only be identified with this equipment. Recently, Schubert and Migliaccio [8] found that the angular vestibuloocular reflex (aVOR) is stable over repeated test sessions when examined using canal plane head impulses using the scleral search coil technique.

Since the beginning of the 1990s, VOR adaptation has been attempted by repeating head movements on one plane from side to side while the patient fixes his eyes on a letter or a point at a given distance. This exercise, which is known as paradigm *x*1, is repeated for one or two minutes from three to five times per day. In addition, the *x*1 viewing exercises are often performed with vertical head movements [9, 10].

Initially, our female patient suffered from chronic vestibular hypofunction, with minimal and fluctuating changes in dynamic visual acuity and her perception of handicap even after having participated in several months of vestibular rehabilitation. She did not experience an improvement until we changed her treatment by adding a unique VOR exercise that asked her to make ipsilesional, high frequency, and low amplitude head impulses. The head rotation was only done towards the affected ear, while the patient used the vHIT equipment concurrently. Here we report the changes observed in the results of the vHIT, the dynamic visual acuity clinical test (DVA), and the perception of handicap after unilateral training with vHIT equipment.

## 2. Case Report

**Figure 1:**
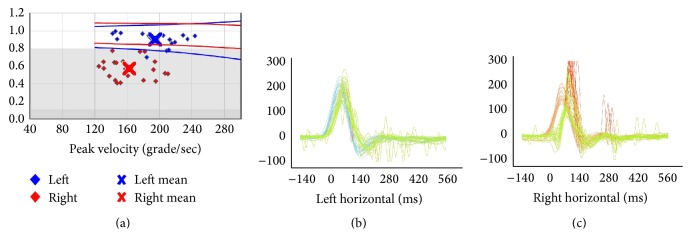
Horizontal passive vHIT before unilateral training: left gain: 0.90 ± 0.08; right gain: 0.57 ± 0.11; asymmetry: 37%.

A young female, 30 years old, presented in our clinic suffering a right side unilateral vestibular hypofunction (UVH) due to vestibular neuritis (VN) [11]. At the time of diagnosis, she reported sudden and severe rotatory vertigo with associated autonomic symptoms over the previous 48 hours. Pure tone audiometry (PTA) in both ears was 5 dBHL; she had no tinnitus. The patient denied any previous history of related problems, although her mother was suffering from migraine headache. The patient was hospitalized three days, where she received intravenous steroids and antiemetics. Inner ear and cerebellar magnetic resonance imaging (MRI) were normal. She began vestibular rehabilitation treatment, with a progressive exercise regimen. Initially, she performed gaze stability and balance exercises 3–5 times a day in her home, for a total stimulus time of 20–40 min daily. The gaze stability exercises included the *x*1 and *x*2 paradigms for both near and far target distances. The balance exercises were provided to improve her use of vestibular information to maintain balance. We progressed these exercises by reducing the base of support, altering vision, and proprioceptive input (eyes open or closed; standing on a firm or soft surface). The gait exercises included walking in tandem, with eyes closed, with cephalic movement in the sagittal and horizontal planes. Habituation exercises were indicated based on the result of the 16 movements from the Motion Sensitivity Quotient [12]. The habituation movements included 4 repetitions, 4 times a day, until the exercises did not generate any symptoms for 48 hours, at which time the patient suspended them. Videonystagmography (VNG) showed unilateral weakness of the right ear at 78%. At the time of discharge, her Dizziness Handicap Inventory (DHI) was 66. The patient continued with this rehabilitation treatment for 9 months, after which the final DHI improved to 36. Although she said that she did all the prescribed exercises, she reported blurred vision and a permanent dizzy sensation. She decided to discontinue treatment but returned to the clinic after three months with the same symptoms. Additionally, she now reported a new onset of periodic headaches that did not fulfill migraine or vestibular migraine criteria [13]. A repeated VNG exam showed spontaneous nystagmus towards the left, with a slow phase velocity (SPV) of 7°/sec. At that moment DHI was 54; the Motion Sensitivity Quotient (MSQ) was 11.81 points; Functional Gait Assessment (FGA) was normal; and modified Clinical Test of Sensory Interaction and Balance (mCTSIB) was 120/120. She restarted vestibular rehabilitation and after 10 sessions her DHI was not better (64 points). Laboratory studies showed normal levels for FAN (Antinuclear Factor), folic acid, Anti-DNA, ionogram, magnesium, calcium, proteinogram, VDRL, and vitamin B12. However, she reported that her headaches had become premenstrual and they now fulfilled the criteria for a migraine diagnosis. She was started on 12.5 mg oral amitriptyline daily and prescribed dietary measures. Subsequently this patient reported less blurred vision and dizziness and no headaches for two months. A new MRI and an angiocerebral MRI were normal. Nevertheless, the patient returned two months later complaining of persistent dizziness, blurred vision, and no benefits from the amitriptyline in spite of the fact that the dosage had been raised to 50 mg daily. We modified her treatment to start vestibular rehabilitation again (her initial DHI was 40) and she was started on 25 mg oral topiramate daily. The patient did not tolerate this medication and discontinued its use. Next, she was prescribed 10 mg of flunarizine daily with good tolerance. Repeated VNG showed spontaneous left nystagmus with a SPV of 3°/sec. Her DHI was now 34, but her clinical dynamic visual acuity was abnormal showing a 6-line difference from static visual acuity. During the next 4 months, the patient did not come to the clinic, after which period of time she came in and was treated with 25 mg of oral venlafaxine daily (provided by another clinic). At this point, we tested with the vHIT (ICS impulse 1085 Otometrics®), which showed a gain of 0.57 in the right horizontal canal with overt and covert saccades (Figure 1). The other semicircular canals had normal gains. Electrocochleography was normal, Brainstem Auditory Evoked Response was normal, a new MRI was normal, and PTA was normal. A 4th VNG showed spontaneous horizontal nystagmus to the right, with a SPV of −3°/sec (was interpreted as a recovery nystagmus), and an orthoptic evaluation was normal. Though she said she still suffered from blurred vision and dizziness, she reported fewer headaches. We prescribed 24 mg daily of betahistine (8 mg every 8 hours) to improve compensation. While the patient experienced an improvement, she reported that the dizziness persisted throughout the day. She began working again but had to stop 1 month later due to blurred vision, headaches, and dizziness. Once again, she began vestibular rehabilitation plus psychological therapy. This combination of therapies resulted in some improvement, though her symptoms persisted during head movement, especially in the dark. Her constant blurred vision made it difficult for her to read. Her 5th VNG showed that a spontaneous nystagmus to the left was 1.8°/sec, while the vHIT was the same as before. Based on persistent symptoms and lack of improvement with traditional vestibular rehabilitation, we elected to treat her using passive and predictive, yaw head impulses towards the affected side only. This was done for five consecutive days. Video HIT was used to ensure that the velocity of impulse was correct to stimulate her right horizontal canal in the field of fast movements. When the patient did the head impulse exercises, she sat in front of a solid black circle of 10 mm in diameter on a white background placed at one meter. This circle was positioned at the same level or height as her occipitonasal axis. Stimulation of the affected semicircular canal was done with 10 series of 15 passive head impulses (done by the therapist) with 30 seconds of rest between each series. The initial head position was such that the patient's gaze was centered on the point in front of her with ±2° between the horizontal and vertical planes [14]. The head impulses were small and fast, with a peak amplitude of 15 degrees, a peak velocity of 150°/sec, and peak acceleration of 3000°/sec; the return to the initial position was slow. Video HIT equipment was used to monitor the velocity and amplitude of movements, which were corrected when necessary.

**Figure 2:**
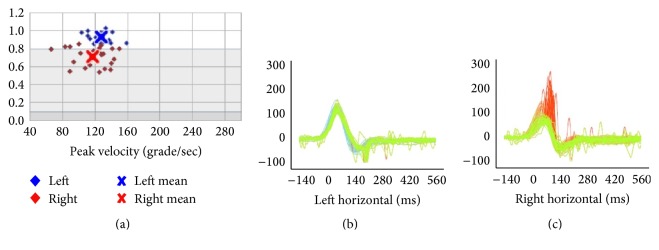
Horizontal passive vHIT after unilateral training: left gain: 0.93 ± 0.05; right gain: 0.71 ± 0.10; asymmetry: 24%.

After this treatment, the patient reported resolution of blurred vision and dizziness, with no premenstrual migraines or vertigo. Her final vHIT showed a gain of 0.71 for the right horizontal semicircular canal with covert saccades (Figure 2). The VOR gain remained normal in the vertical canals. Her final DHI was 12. The clinical horizontal dynamic visual acuity test was now within 2 lines of her static visual acuity (normal). After 6 and 12 months, she returned for follow-up to report that she had no more vestibular symptoms. Her premenstrual migraines persisted and she continued to have a spontaneous nystagmus beating towards the left at 1.8°/sec SPV, in the dark (Table 1).

## 3. Discussion

Today there is moderate to strong evidence that vestibular rehabilitation is a safe and effective treatment for patients with unilateral peripheral vestibular disorders [15, 16]. However, the evidence on frequency, intensity, and time, as well as details on vestibular rehabilitation (for example, in compensation exercises) is still limited, due in part to the heterogeneity of research papers [15]. The objectives of vestibular rehabilitation include a reduction in dizziness and in the risk of falls, increased confidence in equilibrium, and better VOR function [17, 18]. In 2012, Herdman et al. [19] did a study on the possible variables that could affect the results of vestibular rehabilitation and found that patients with greater loss of vestibular function were less likely to return to normal DVA following a course of vestibular exercises, although they still showed significant improvement. In our own clinical practice, we have observed that some patients do not achieve normal DVA in spite of doing the *x*1 paradigm exercises daily. Our observation leads us to think that patients with unilateral vestibular hypofunction who do these exercises at home might not be moving their heads with the appropriate velocity or amplitude in order to avoid the sensation of dizziness and blurred vision caused by the retinal slip. We therefore suspect that the lack of improvement in our patient during the traditional vestibular rehabilitation was due to errors of execution. Asymmetry in vestibular function can cause oscillopsia, which is a sensation of blurred vision during head rotation. In patients with UVH, this can occur during ipsilesional head rotations [20–22]. In one study [23], done on monkeys subjected to a unilateral labyrinthectomy, the authors explain that since “in everyday activity the animal moves its head in both directions and never repeatedly in one direction, there may be a conflict in the error signal induced by motion in the contralesional and ipsilesional directions. This error signal could result because the gain is normal for rotations in the contralesional direction. Therefore, an increase in gain would cause an error signal opposite to the error signal resulting from the low gain in the ipsilesional direction. Rotating the animal exclusively in one direction overcomes this limitation because the animal receives only an error signal to increase the gain.” The fundamental finding of this study was that asymmetry in the VOR gain after unilateral labyrinthectomy did not improve until the monkeys received ipsilesional adaptation training. The exercises our patient did were different from those performed by the monkeys in that our patient did head only ipsilesional rotation, not whole-body ipsilesional rotation.

We have compared our results with those of other studies done on humans. Schubert et al. [24] have studied unilateral VOR training using an incremental visual stimulus and measured VOR gain with scleral search coil system. Unlike our study, active head impulses only were used in ten series of 15 stimuli towards each side, and the laser was activated only when the head was moved towards the side where adaptation was desired. The laser was gradually adjusted by increments of ten percent of the head movement velocity, until reaching 100 percent in the last series. The range of movement (15°), the velocity (150 m/s), and the acceleration (3000° m/s^2^) were the same as those we used for our patient. The authors found that adaptation to unilateral stimuli was possible in healthy subjects. Measurements were taken for both active and passive head rotation, even though the subjects were trained using active only head rotation. The gain in the VOR towards the adaptive side after training increased by 22% with active head movements and 11% with passive head movements. In a recent pilot study done by the same authors [25] on ten subjects (six controls and four patients with unilateral and bilateral vestibular hypofunction), active and passive VOR gains were measured during high acceleration stimulation, before and after training for unilateral VOR adaptation, using a helmet with a laser and a gyroscope. VHIT equipment like that used with our patient was used to measure gains in the VOR and it was found that these improved with both active and passive head impulses in patients with unilateral and bilateral hypofunction, though, given the small number of patients, these results were not statistically significant. The exercise variant we had our patient do was different from those used in these studies, in that we used a fixed point as in the *x*1 paradigm described by Herdman. Additionally, we did not use an incremental stimulus, nor did we use a helmet with an attached laser. Instead, in our study the head impulses were performed by the patient with chronic vestibular hypofunction.

We found it useful to know the VOR gain and presence of saccades as provided by the vHIT software, during our training. We believe that knowing this information was helpful and further believe that vHIT might be useful to assist other clinicians in training the VOR [24, 25].

One limitation of our study is the difference in passive head velocity used to measure VOR gain between the pre- and postmeasures. The lower head velocity for the postmeasure would not be expected to inhibit the contralesional afferents. However, the covert and overt saccades did still change as evidence by their more consistent latency. Another weakness of our study is our lack of a control subject, which limits the ability to make strong conclusions. However, we feel that our results suggest that, after 5 consecutive daily sessions, the VOR gain increased, the covert saccades were reprogrammed, and vHIT asymmetry was reduced. Additionally, gaze instability as measured by DVA and her perception of handicap both improved.

The vHIT and the caloric test of the VNG are known to present with different responses of the VOR, presumably due to stimulating the VOR at different frequencies. Redondo-Martínez et al. [26] found no correlation between the VHIT, the caloric test, and the DHI test in patients with vestibular neuritis, none of which were indicative of the subjective clinical status of the patient at any given time. In line with recent scientific literature [26–28], our patient showed improved DHI and DVA without significant changes in her vHIT. One study [29] has demonstrated that persistent dizziness after VN is not significantly associated with sustained vestibular impairment as assessed by the quantitative search coil head impulse testing (qHIT); more specifically, severe vestibular deficit in the chronic patient group did not imply a high score on the shortened version of the Vertigo Symptom Scale (sVSS), assessing dizziness, vertigo, and imbalance during the past 12 months. Similar findings were reported in a study [30] on patients who suffered from vestibular neuritis; the high velocity VOR was not different between patients who felt they had recovered and patients who felt they had not and suggests that chronic symptoms of dizziness following VN are not associated with the high velocity VOR of the single or combined ipsilesional horizontal and anterior or posterior semicircular canals.

To better understand the possible benefits of these exercises, other studies should be done in patients with unilateral and bilateral vestibular hypofunction to compare the *x*1 paradigm exercises with our novel, ipsi-rotational exercises. A recent review [31] concluded that investigations would be required to determine the evolution of the VOR gain with the progression of the vestibular disease. We also recommend further evaluation of the use of active or passive head movements and the relation between these exercises and different variations of them as well as evaluation of the possibility of performing this exercise at patients' homes without expensive equipment.

## 4. Conclusion

Passive unilateral head impulses applied to the affected side appear to be a useful method for stimulating recovery of gaze stabilization in our subject with unilateral vestibular hypofunction and abnormal DVA. Larger studies are needed to evaluate the efficacy of this exercise.

## Figures and Tables

**Table 1:** Evolution of the symptoms, diagnosis, and treatment.

June 2011	2011–2014	July 2014	August 2014	November 2016
Initial symptoms	Evolution	Before treatment with unilateral head impulses	After treatment with unilateral head impulses	Phone communication
Sudden rotational vertigo lasting more than 48 hours, intense autonomic symptoms	Beginning of menses migraine	Migraine, less intensity and frequency	Premenstrual migraine	Sporadic migraine
Unsteadiness +++	Unsteadiness ++	Unsteadiness ++	Unsteadiness +	Unsteadiness +
Nausea and vomiting	Blurred vision with head movements +++	Blurred vision with head movements +++	Blurred vision with head movements +	Blurred vision with head movements ++
		DVA 6 lines (abnormal)	DVA 2 lines (normal)	
	DHI: 66	DHI: 34	DHI: 12	
	Dizziness ++	Dizziness ++	Dizziness +	Dizziness +
	Dizziness in darkness ++	Dizziness in darkness ++	Dizziness in darkness +	Dizziness in darkness ++
Unilateral weakness right ear 78% (VNG)		Unilateral weakness right ear 80% (VNG)		
Diagnosis: vestibular neuritis	Diagnosis: right vestibular hypofunction without compensation. Premenstrual migraine	Diagnosis: right vestibular hypofunction without compensation. Premenstrual migraine	Diagnosis: right vestibular hypofunction with compensation. Premenstrual migraine	Diagnosis: premenstrual migraine. The patient should do new clinical and lab evaluation
Vestibular rehabilitation	Vestibular rehabilitation and migraine prophylaxis	Betahistine plus unilateral head impulses	No treatment	Waiting for new evaluation

+++: severe; ++: moderate; +: mild; DVA: dynamic visual acuity; VNG: videonystagmography; DHI: Dizziness Handicap Inventory.
